# Precise genetic mapping and integrative bioinformatics in Diversity Outbred mice reveals *Hydin* as a novel pain gene

**DOI:** 10.1007/s00335-014-9508-0

**Published:** 2014-04-05

**Authors:** Jill M. Recla, Raymond F. Robledo, Daniel M. Gatti, Carol J. Bult, Gary A. Churchill, Elissa J. Chesler

**Affiliations:** 1 IGERT Program in Functional Genomics, Graduate School of Biomedical Sciences and Engineering, The University of Maine, Orono, ME 04469 USA; 2 The Jackson Laboratory, 600 Main Street, Bar Harbor, ME 04609 USA

## Abstract

Mouse genetics is a powerful approach for discovering genes and other genome features influencing human pain sensitivity. Genetic mapping studies have historically been limited by low mapping resolution of conventional mouse crosses, resulting in pain-related quantitative trait loci (QTL) spanning several megabases and containing hundreds of candidate genes. The recently developed Diversity Outbred (DO) population is derived from the same eight inbred founder strains as the Collaborative Cross, including three wild-derived strains. DO mice offer increased genetic heterozygosity and allelic diversity compared to crosses involving standard mouse strains. The high rate of recombinatorial precision afforded by DO mice makes them an ideal resource for high-resolution genetic mapping, allowing the circumvention of costly fine-mapping studies. We utilized a cohort of ~300 DO mice to map a 3.8 Mbp QTL on chromosome 8 associated with acute thermal pain sensitivity, which we have tentatively named *Tpnr6*. We used haplotype block partitioning to narrow *Tpnr6* to a width of ~230 Kbp, reducing the number of putative candidate genes from 44 to 3. The plausibility of each candidate gene’s role in pain response was assessed using an integrative bioinformatics approach, combining data related to protein domain, biological annotation, gene expression pattern, and protein functional interaction. Our results reveal a novel, putative role for the protein-coding gene, *Hydin*, in thermal pain response, possibly through the gene’s role in ciliary motility in the choroid plexus–cerebrospinal fluid system of the brain. Real-time quantitative-PCR analysis showed no expression differences in *Hydin* transcript levels between pain-sensitive and pain-resistant mice, suggesting that *Hydin* may influence hot-plate behavior through other biological mechanisms.

## Introduction

Human pain sensitivity varies widely between individuals, and the significant influence of genetic factors on pain sensitivity is now widely appreciated (Mogil et al. 1999a). Genetic differences in underlying nociceptive and central pain processing mechanisms are partially responsible for observed variation in pain sensitivity. Although acute and chronic pain are considered clinically distinct phenomena, they are understood to exhibit some degree of overlap at the physiological and molecular genetic levels (Mogil et al. 1999b, 2005b). A genetic understanding of variability in pain sensitivity is essential to developing improved prevention and treatment methods for both acute and chronic pain.

The application of complex trait analysis to human pain genetics research has facilitated the discovery of genes that underlie variability in pain sensitivity and analgesic response (Chou et al. 2006; Compton et al. 2003; Diatchenko et al. 2005, 2006; Fillingim et al. 2005; Indo et al. 1996; Janicki et al. 2006; Klepstad et al. 2004; Nackley et al. 2006; Poulsen et al. 1996; Rakvag et al. 2005; Reyes-Gibby et al. 2007; Sindrup et al. 1990; Tsao et al. 2011; Zubieta et al. 2003). However, human complex trait analyses typically require tens of thousands of individuals to achieve adequate statistical power, in part due to the inherent difficulty of controlling gene–environment interactions in human cohorts (Flint and Eskin 2012). Control over environmental variables is of paramount importance in genetic studies of complex traits. Even when adequate sample size and statistical power are present, the sum of the identified genetic effects in human genetic studies of complex traits comprises only a fraction of the estimated trait heritability, usually less than half (Stranger et al. 2011). Inbred mouse strains facilitate partitioning of trait variance into genetic and environmental components—a nearly impossible task in human studies of pain (Mogil and Grisel 1998). Pain genetics studies using mouse models require only a few hundred animals to identify loci that explain 50 % or more of the phenotypic variance for a particular trait (Flint and Eskin 2012).

Genetic linkage mapping, a technique commonly used to map regions of the genome associated with a phenotype of interest, has facilitated the identification of at least 14 pain-related quantitative trait loci (QTL) in the laboratory mouse to date (Devor et al. 2005, 2007; Furuse et al. 2003; Honda and Takano 2009; LaCroix-Fralish et al. 2009; Mogil et al. 1997, 2005b, 2006; Nair et al. 2011; Nissenbaum et al. 2010; Seltzer et al. 2001; Wilson et al. 2002). Most current mapping populations are produced using traditional two-strain breeding schemes, resulting in QTL spanning an average of ~30 Mbp and containing hundreds of potential candidate genes. The low genetic recombination density and resulting lack of mapping precision afforded by historical mapping crosses necessitates years of additional fine mapping to elucidate the underlying candidate gene(s). Identification of the *Pain1* candidate gene *Cacng2* (MGI:1316660; calcium channel, voltage-dependent, gamma subunit 2) involved 3 years of additional fine mapping by three different laboratories (Devor et al. 2005, 2007; Nissenbaum et al. 2010; Seltzer et al. 2001). The 50 Mbp thermal pain QTL *Tpnr4* required the generation of two F2 hybrid crosses and a congenic strain to identify the candidate gene *Calca* (MGI:2151253; calcitonin/calcitonin-related polypeptide, alpha) (Mogil et al. 2005b). In the case of *Mc1r* (MGI:99456; melanocortin 1 receptor), narrowing of the original 24 Mbp QTL and confirmation of its female-specific role in pain response required an additional 2 years of work (Mogil et al. 2003, 2005a).

Emerging mouse populations such as the Diversity Outbred (DO) stock (Churchill et al. 2012) offer increased heterozygosity and allelic diversity compared to conventional mapping populations. DO mice are produced by the repeated random outcrossing of non-siblings from the Oak Ridge National Laboratory (ORNL) Collaborative Cross (CC) colony (Chesler et al. 2008), a genetically defined and reproducible panel of recombinant inbred strains derived from a set of eight genetically diverse parental strains (A/J, C57BL/6J, 129S1/SvImJ, NOD/ShiLtJ, NZO/HlLtJ, CAST/EiJ, PWK/PhJ, and WSB/EiJ). The DO mouse population captures the same set of natural allelic variants derived from a common set of eight founder strains. Due to a randomized outbreeding strategy, each mouse is a unique individual with one of an effectively limitless combination of the segregating alleles. DO mice have approximately 45 million segregating SNPs—four times the number found in classical laboratory mouse strains—making them an ideal resource for high-resolution genetic mapping (Svenson et al. 2012).

**Fig. 1:**
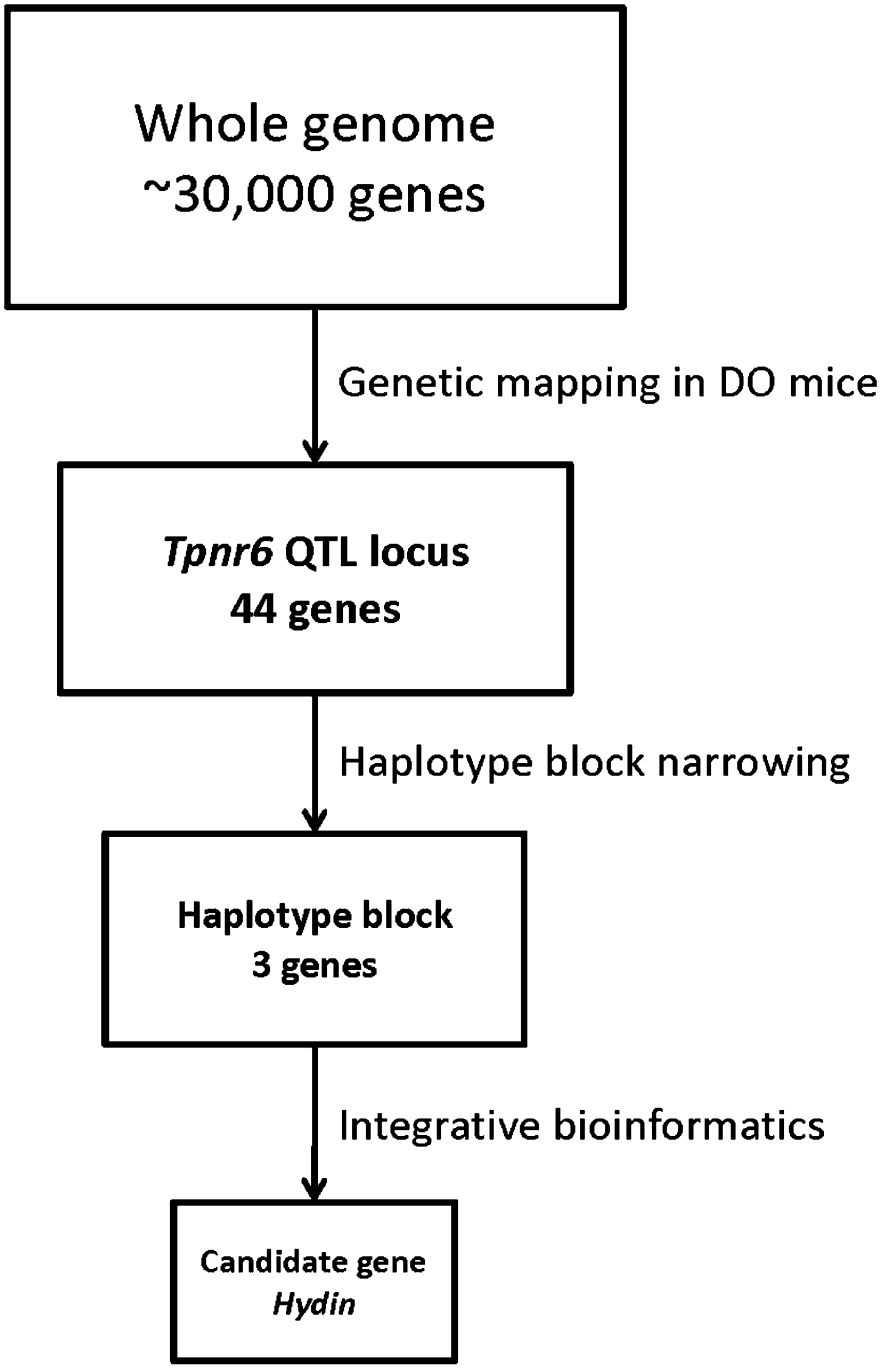
Study Workflow. The above illustration depicts the power of precise genetic mapping combined with integrative bioinformatics to elucidate candidate genes involved in complex traits. The application of genetic and genomic techniques facilitated the quick identification of a single candidate gene for hot-plate response, the protein-coding gene, *Hydin*

We present here the first application of DO mice to pain genetics research. Utilizing a statistical algorithm recently developed for genetic linkage mapping in CC and DO mice (Svenson et al. 2012), we were able to map a 3.8 Mbp QTL containing 44 candidate genes in a single study using ~300 DO mice. We narrowed the QTL region, which we have tentatively named *Tpnr6* (thermal pain response 6), by partitioning the QTL into intervals using haplotype blocks. The narrowed *Tpnr6* region is ~230 Kbp in size and contains three putative candidate genes: (1) the protein-coding gene *Hydin* (axonemal central pair apparatus protein; MGI:2389007), (2) the lincRNA gene *Gm26832* (predicted gene, 26832; MGI:5477326), and (3) the snRNA gene *Gm23627* (predicted gene, 23627; MGI:5453404). We assessed the plausibility of each candidate gene’s role in pain response using an integrative bioinformatics approach, combining data related to protein domain, biological annotation, gene expression pattern, and protein functional interaction. Our results reveal a novel, putative role for the protein-coding gene *Hydin* in thermal pain response, possibly through its role in ciliary motility in the choroid plexus–cerebrospinal fluid (CP–CSF) system of the brain (Fig. 1).

## Materials and methods

### Diversity Outbred mice

Incipient CC breeding lines used to establish the DO population at The Jackson Laboratory were received from ORNL and were of generation G2:Fn (*n* ≤ 12). Male and female DO mice (*n* = 283; J:DO, JAX stock number 009376) from generations 4 and 5 (G4 and G5) of outcrossing were obtained from The Jackson Laboratory at 6 weeks of age and transferred to the housing facility via wheeled cart. Mice were acclimated to the vivarium for at least 2 weeks prior to testing at 13–17 weeks of age. Mice were housed in duplex polycarbonate cages with a Shepherd Shack on ventilated racks providing 99.997 % HEPA filtered air to each cage in a climate-controlled room under a standard 12:12 light–dark cycle (lights on at 0600 h). Pine cob bedding was changed weekly and mice were provided ad libitum access to food (NIH31 5K52 chow, LabDiet/PMI Nutrition, St. Louis, MO, USA) and acidified water. Initially, all mice were housed in a cage density of five males or females. During the course of the study, ∼20 % of G4 and 46 % of G5 pens of male mice were separated into smaller groups (1–4) due to aggressive behaviors. All procedures and protocols were approved by The Jackson Laboratory Animal Care and Use Committee (Protocol #EJC10-06), and were conducted in compliance with the National Institutes of Health Guidelines for the Care and Use of Laboratory Animals.

### Genotyping

DNA was prepared from tail biopsies and genotyping was outsourced to GeneSeek (http://www.neogen.com/GeneSeek) for analysis using the Mouse Universal Genotyping Array (MUGA; GeneSeek, Lincoln, NE, USA). Built on the Illumina Infinium platform (San Diego, CA, USA), the MUGA contains 7,851 SNP markers distributed throughout the mouse genome (average spacing of 325 Kb, standard deviation of 191 Kb) (Neogen 2010). The array’s SNP panel was optimized for the eight laboratory mouse strains that are founders of the DO mapping population, making the MUGA optimal for detecting heterozygous regions and discriminating between haplotypes in homozygous regions (Neogen 2010). The MUGA marker panel provides an average effective sampling sensitivity of just over 1 Mb, allowing any of the CC founders to be identified within a window of four to five SNPs.

### Experimental design

A total of 283, 13–17 week old, DO mice (139 male, 144 female) were phenotyped using the hot-plate assay of acute thermal nociception. The DO mice utilized in this study were part of a larger phenotyping initiative described by Logan et al. (2013), in which the mice were subject to a battery of noninvasive behavioral tests to assess activity, anxiety, and response to novelty. Assays were performed in order of increasing perceived stressfulness in an effort to minimize the impact of carry-over effects as follows: day 1, open-field; day 3, light–dark box; day 4, visual-cliff avoidance; day 5, hot-plate; and day 9, tail-suspension test. Mice were randomly assigned to testing groups, such that an equal number of male and female mice were tested each day (*n* = ~24 per sex). Groups 1 through 4 were comprised of G4 individuals; groups 5 and 6 were of generation G5. Mice were between 12 and 16 weeks of age on the first day of testing. For the open-field, light–dark box, and visual-cliff tests, mice were habituated to the testing room for 1 h prior to testing, and 30 min was used for the hot-plate and tail-suspension tests. For each assay, mice were removed by the tail then returned to the clean side of a duplex home cage until each cage mate had completed testing. Several experimenters participated in the testing, but a single experimenter handled the mice for each test and the same individuals were in the room during all sessions of a particular test.

### Hot-plate assay of acute thermal nociception

A modified version of the technique of Eddy and Leimbach (1953) [originally described by Woolfe and MacDonald (1944)] was used to perform the hot-plate assay. Mice were brought to the testing room at 9:00 a.m. and allowed to acclimate for 30 min. Pain reflexes in response to a thermal stimulus were measured using a Hot Plate Analgesia Meter (IITC Life Science Inc., cat #39, Woodland Hills, CA, USA) as previously described (Bannon and Malmberg 2007; Mogil et al. 1999a; Wilson and Mogil 2001). The surface of the hot plate (11 cm × 10.5 cm) was preheated to a constant temperature of 55 °C. Each mouse was individually placed on the hot plate surrounded by a clear acrylic cylinder (15 cm tall, 10 cm internal diameter, open top with clear acrylic lid) to constrain locomotion of the subject. Each mouse remained on the plate until it performed either of two behaviors regarded as indicative of nociception: hindpaw lick or hindpaw shake/flutter (Espejo and Mir 1993; Hammond 1989). Latency to respond to the heat stimulus was measured to the nearest 0.01 s. Each mouse was immediately removed from the hot plate as soon as it performed either of the two nociceptive responses. If a mouse did not respond within 30 s, the test was terminated and the mouse removed from the hot plate. Animals were tested individually and were not habituated to the apparatus prior to testing. Only hindpaw responses were used as endpoint criteria, as forepaw licking and lifting are components of normal grooming behavior (Mogil et al. 1999a). Jumping was not included as a phenotypic endpoint due to uncertainty as to whether jumping behavior is indicative of a nociceptive or activity-related response. Logan et al. (2013) noted that the inclusion of wild-derived alleles in the DO can result in atypical or “inappropriate” behaviors in a subset of mice during certain behavioral tests. While behaviors such as repeated jumping, wall climbing, and alternate paw lifting are interesting variants amenable to genetic mapping, they require careful analysis and interpretation and were not included as part of this study. Fourteen of the 283 phenotyped mice were excluded from the genetic linkage mapping analysis because they exhibited such behaviors.

### Genetic linkage mapping

Eight DO samples were excluded from mapping analysis due to missing genotype calls, bringing the total number of mice to 261. QTL mapping was carried out as described by Svenson et al. (2012). Founder haplotypes were reconstructed using a Hidden Markov Model (HMM) that produced a matrix of 36 genotype probabilities for each sample at each SNP. Genotype probabilities at each SNP were then collapsed to an eight-state allele dosage matrix by summing the probabilities contributed by each founder. Phenotypic data were normalized by natural log transformation prior to linkage mapping analysis. Mapping was performed using QTLRel software (http://www.palmerlab.org/software) (Cheng 2011). A mixed model was fit with sex and experimental group as additive covariates and a random effect was included to account for kinship. Regression coefficients for additive effects of founder alleles were estimated at each genomic location. Significance thresholds were obtained by performing 1,000 permutations of the genome scans with phenotype data being shuffled among individuals and 2-logarithm of odds (LOD) support intervals from the linear model were determined for significant (*p* ≤ 0.05), suggestive (*p* ≤ 0.10), and trending (*p* ≤ 0.63) QTL peaks. All phenotype and genotype data have been made publicly available through the Mouse Phenome Database (MPD, http://phenome.jax.org/; MPD:470) and the QTL Archive (http://qtlarchive.org/; Recla_2013).

### Identification of candidate genes

The genomic elements (e.g., genes, transposable elements, intergenic regions) associated with the QTL detected by genetic linkage mapping were identified using the MGI mouse genome browser (http://gbrowse.informatics.jax.org/cgi-bin/gbrowse/mouse_current/; NCBI Build 37; analysis conducted August 2011). All genes within the QTL (coding and non-coding) were considered putative candidate genes. SNPs within each gene were examined for the presence of at least one inter-strain polymorphism matching the allelic effect pattern of the genetic mapping results (Churchill et al. 2012). SNP data were obtained from dbSNP (Build 128, http://www.ncbi.nlm.nih.gov/projects/SNP/) and the Wellcome Trust Sanger Institute (http://www.sanger.ac.uk/cgi-bin/modelorgs/mousegenomes/snps.pl).

### Plausibility analysis of candidate genes

In order to begin to explore and understand the biological processes and molecular pathways underlying a complex trait such as pain sensitivity, it is important to understand that while proteins carry out their functions by interacting with each other, they do not necessarily interact physically (Doerr 2010). The integration of heterogeneous genome-wide data can facilitate the development of functional relationship networks revealing the collaborative roles between proteins in carrying out a biological process (Guan et al. 2008). Genes involved in a complex trait are likely to participate in similar or overlapping pathways and should thus be more likely to share protein domains, biological annotations, and patterns of gene expression. Using these paradigms, the plausibility of each candidate gene’s role in pain response was assessed by compiling heterogeneous genomic evidence from a variety of electronic databases.

In order to examine the known functional relationships between each candidate gene and known pain genes, a list of experimentally validated pain-related genes was compiled by querying the online databases described in Table 1 using the terms “pain” and “nociception.” Ingenuity Pathway Analysis (IPA, http://www.ingenuity.com/), a bioinformatics system based on a manually curated knowledgebase, was used to map the known IPA functional interactions between candidate and known pain genes.

**Table 1:** Electronic databases queried for experimentally validated pain-related genes using the keywords “pain” and “nociception”

Resource^a^	Organism	URL
MGD	Mouse	http://www.informatics.jax.org/
PainGenesdb	Mouse	http://www.jbldesign.com/jmogil/enter.html
OMIM	Human	http://www.ncbi.nlm.nih.gov/omim/
SNPs3D	Human	http://www.snps3d.org
RGD	Rat	http://rgd.mcw.edu

^a^Resource abbreviations: *MGD* Mouse Genome Database, *OMIM* Online Mendelian Inheritance in Man, *SNPs3D* Single Nucleotide Polymorphisms—3 Databases (Modules), *RGD* Rat Genome Database

MouseMap (http://sonorus.princeton.edu/mousemap/applet.shtml) is a web-based tool that provides access to tissue-specific predicted functional relationships between mouse genes (Guan et al. 2012). A list of brain ventricular zone functional relationships predicted by MouseMap was obtained from Dr. Yuanfang Guan (University of Michigan Medical Center, Ann Arbor, Michigan) and analyzed for direct relationships with the candidate gene *Hydin* with edge weight scores ≥0.9. The functional relationship networks generated by MouseMap and IPA were assessed for enrichment of Gene Ontology (GO) (Ashburner et al. 2000) and Mammalian Phenotype (MP) (Smith and Eppig 2009) terms using VLAD (http://proto.informatics.jax.org/prototypes/vlad-1.0.3/), a web-based tool for statistical term enrichment (annotation date: November 2, 2011).

For each candidate gene, gene expression annotations were collected from the Allen Brain Atlas (Lein et al. 2007), Gene Expression Omnibus (GEO) (Barrett et al. 2013), IPA, and the Mouse Genome Database (MGD) (Eppig et al. 2012). Serial Pattern of Expression Levels Locator [SPELL (Hibbs et al. 2007)], a query-driven search engine for large gene expression microarray compendia, was used to predict genes that share spatial and temporal expression patterns with the candidate genes. InterPro protein domain (Hunter et al. 2012) and MP annotations were obtained from MGD. Pain-related phenotype data were collected from MGD, IPA, PainGenesdb, and GeneWeaver, a web-based tool that allows users to integrate phenotype-centered gene sets across species, tissues, and experimental platform (Baker et al. 2012). GO terms were obtained from MGD and AmiGO (Carbon et al. 2009), and IPA canonical pathways were obtained from IPA. All queries were performed July–November of 2011. A summary of the electronic databases mined for biological annotations during this analysis is provided in Table 2.

**Table 2:** Electronic databases queried for biological annotations during candidate gene plausibility analysis

Resource^a^	Annotation(s) provided	URL
ABA	Gene expression	http://mouse.brain-map.org/
GEO	Gene expression	http://www.ncbi.nlm.nih.gov/geo/
SPELL	Predicted co-expression with candidate genes	http://spell.jax.org/
AmiGO	Gene Ontology term	http://geneontology.org/
GeneWeaver	Pain-related phenotype	http://www.geneweaver.org/
PainGenesdb	Pain-related phenotype	http://www.jbldesign.com/jmogil/enter.html
IPA	Expression	http://www.ingenuity.com/products/pathways_analysis.html
	Pain-related phenotype	
	IPA canonical pathway	
MGD	Expression	http://www.informatics.jax.org/
	InterPro protein domain	
	Pain-related phenotype	
	Gene Ontology term	
	Mammalian Phenotype term	

^a^Resource abbreviations: *ABA* Allen Brain Atlas, *GEO* Gene Expression Omnibus, *SPELL* Serial Pattern of Expression Levels Locator, *IPA* Ingenuity Pathway Analysis, *MGD* Mouse Genome Database

### Real-time quantitative PCR analysis of the candidate gene *Hydin*

Whole brains from male and female pain-sensitive (C57BL/6J) and pain-resistant (A/J) mice (*n* = 5/group) were dissected and stored in RNA*later* ^®^ (Life Technologies, Carlsbad, CA, USA) prior to homogenization in TRIzol^®^. Total RNA was extracted using the PureLink™ RNA Mini Kit (Life Technologies, Carlsbad, CA, USA) as per manufacturer’s protocol. Samples were further purified with on-column DNase I treatment (Qiagen, Venlo, Netherlands). Extracted RNA samples were assessed for integrity and concentration using the Agilent Bioanalyzer (Agilent Technologies, Santa Clara, CA, USA) and Nanodrop (ThermoFisher Scientific, Waltham, MA, USA), respectively. Samples with RNA integrity numbers (RIN) of 7 and above were used for downstream applications. cDNA was synthesized from extracted RNA using the MessageSensor™ RT Kit (Life Technologies, Carlsbad, CA, USA). *Hydin* expression levels were measured using diluted cDNA with the TaqMan Gene Expression system (Life Technologies, Carlsbad, CA, USA) and the Applied Biosystems Prism 7900HT real-time quantitative PCR system (Life Technologies, Carlsbad, CA, USA) with a pre-designed target candidate gene assay (Mm00556723_m1; Life Technologies, Carlsbad, CA, USA). Cycle conditions consisted of 2 min at 50 °C and 10 min at 95 °C, followed by 40 cycles of 15 s at 95 °C, 1 min at 60 °C. Relative fold change in expression was normalized to *Gapdh* expression using the well-established delta–delta Ct method (Livak and Schmittgen 2001). All assays were performed in triplicate. *Hydin* expression level was reported as mean ± SE and a linear regression model was used to investigate the effects of sex, strain, and their interaction (significant differences declared at *p* ≤ 0.05). Statistical comparisons were performed using the JMP 10.0 software package.

## Results

### Genetic linkage mapping

**Fig. 2:**
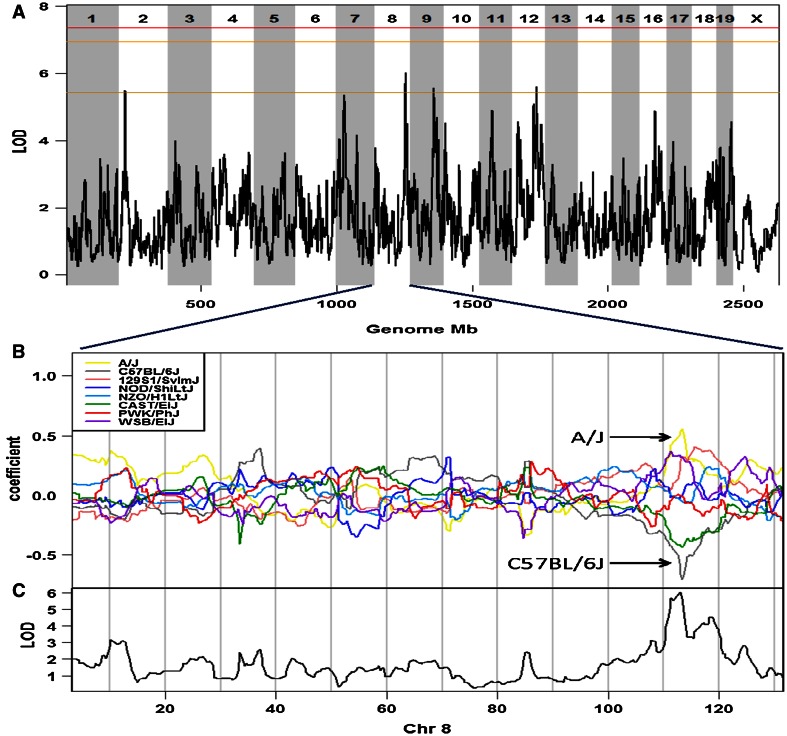
Hot-plate latency has a significant QTL on chromosome 8. **a** Genome-wide scan for hot-plate latency reveals a QTL with a peak LOD score of 6.1. Permutation-derived significance thresholds are marked by *horizontal lines*: 0.63 (*bottom*), 0.1 (*middle*), 0.05 (*top*). **b** The eight coefficients of the QTL model show the effects on hot-plate latency contributed by each founder haplotype on chromosome 8. *Colored lines* represent the phenotypic effect of each DO founder strain’s allelic contribution at each SNP locus across the chromosome. **c** QTL plot for the chromosome 8 locus

Linkage mapping identified one QTL peak nearing genome-wide significance located on chromosome 8, which has been tentatively named thermal pain response 6 [*Tpnr6*; MGI:5294244 (provisional)] (Fig. 2a). The peak of the *Tpnr6* QTL is located at chr8:113017713 (rs31242180, NCBI Build 37) and has a LOD score of 6.1. An approximate “confidence interval” for *Tpnr6* was calculated using a 2-LOD drop, resulting in an interval width of 3.8 Mbp (5′ flanking SNP UNC080383989, Chr 8: 110437369; 3′ flanking SNP rs31329971, Chr 8:114226254).

The allelic effect plot shown in Fig. 2b illustrates the phenotypic effect of each DO founder strain’s genetic contribution at each genomic position along chromosome 8. Within *Tpnr6*, the allelic contribution from A/J is reported to condition high hot-plate latency (low sensitivity to acute thermal pain), while allelic contributions from CAST/EiJ and C57BL/6J are reported to condition low hot-plate latency (high sensitivity to acute thermal pain). The remaining five DO founder strains are reported to have allelic contributions that condition responses to acute thermal pain that fall between A/J and C57BL/6J in the following order:

1. A/J (least sensitive)
2. WSB/EiJ and 129S1/SvlmJ
3. NOD/ShiLtJ and PWK/PhJ
4. NZO/HlLtJ
5. CAST/EiJ and C57BL/6J (most sensitive)

Hot-plate data from inbred DO founder strains have been generated by four independent labs: Jaxwest (2013), Mogil et al. (2013), Koide et al. (2013), and Chesler et al. (2013). All four datasets recapitulate the inbred strain sensitivity ranking predicted by the allelic contributions at the *Tpnr6* locus (the rankings of NOD/ShiLtJ and C57BL/6J are reversed in the Chesler study).

### Candidate gene identification

The 3.8 Mbp *Tpnr6* QTL contains 44 protein-coding and non-coding genes (NCBI Build 37; MGI Genes and Markers query performed July 2011, Feature Type “gene”). We further narrowed the *Tpnr6* interval by identifying haplotype blocks within *Tpnr6* that differ between A/J and C57BL/6J | CAST/EiJ and the remaining five DO founder strains. This method of partitioning QTL into intervals that can be summarized by a single phylogenetic tree among progenitors is analogous to the approach demonstrated by Goodson et al. (2012) using anxiety phenotypes.

We used the University of North Carolina’s Mouse Phylogeny Viewer (MPV, http://msub.csbio.unc.edu) to identify one haplotype block region within *Tpnr6* that differed between A/J and C57BL/6J (Chr 8:112802216.113013057). The MPV does not incorporate haplotype data from the three wild-derived DO founder strains (WSB/EiJ, PWK/PhJ, CAST/EiJ). To circumvent this limitation, we examined SNP data from the Wellcome Trust Sanger Institute (REL-1211; NCBI build 37) and searched for haplotype regions within *Tpnr6* matching the A/J / C57BL/6J | CAST/EiJ allelic effect pattern. We identified a SNP exclusive to A/J near the 5′ end of the haplotype block reported by the MPV, classified by Sanger as “upstream_genetic_variant” and “downstream_genetic_variant” (no rsID; chr8:112787281). We observed several CAST/EiJ-exclusive SNPs within the *Tpnr6* locus, particularly near the 3′ end of the candidate gene *Hydin*. A single CAST/EiJ-exclusive SNP classified as “upstream_genetic_variant” was present near the reported end of the MPV haplotype block (rs245877262; chr8:113018557), just downstream of the 3′ end of *Hydin*. There were also several regions within *Tpnr6* where the C57BL/6J haplotype was distinct from the remaining strains, including large regions between the A/J- and CAST/EiJ-exclusive SNPs. We interpret our observations to be consistent with the allelic effect pattern observed at the *Tpnr6* locus. It is likely that there are at least two SNPs conditioning the observed allelic effect; one private SNP between the five inbred strains (A/J-exclusive), and one private SNP between the inbred strains and CAST/EiJ (CAST/EiJ-exclusive). We used these haplotype data to further narrow the *Tpnr6* locus. The narrowed haplotype region is ~230 kbp wide and contains three putative candidate genes:

1. *Hydin* (axonemal central pair apparatus protein; MGI:2389007); protein-coding gene.
2. *Gm26832* (predicted gene, 26832; MGI:5477326); lincRNA gene.
3. *Gm23627* (predicted gene, 23627; MGI:5453404); snRNA gene.

The narrowed *Tpnr6* interval contains four validated SNPs that differ between A/J and C57BL/6J: rs32704439, rs13479984, rs33400842, rs33170521 according to dbSNP at NCBI and SNP data from the Mouse Genomes program at the Wellcome Trust Sanger Institute. All four SNPs are located within intronic sequence of the protein-coding gene *Hydin*. Two of the SNPs (rs32704439 and rs13479984) are present as the same allelic variant in C57BL/6J and CAST/EiJ, matching the allelic effect pattern indicated by the genetic mapping results (CAST/EiJ data for rs33400842 and rs33170521 not available). Thus, of the three candidate genes present within the narrowed *Tpnr6* locus, only *Hydin* is reported to contain inter-strain polymorphisms that match the effect pattern of our genetic mapping results, suggesting *Hydin* as the most plausible candidate gene.

### Plausibility analysis of candidate genes’ role in pain response

The biological annotations, pathways, and functional interaction networks associated with each of the three *Tpnr6* candidate genes were identified as described in “Plausibility analysis of candidate genes” section. At the time of this writing, there were no biological annotations or functional data for the predicted genes *Gm26832* or *Gm23627*. *Hydin* is known to be expressed exclusively in motile secondary cilia, which are present in only a few distinct anatomical regions in humans and mice: respiratory epithelium, luminal surface of the oviduct, efferent ducts of the testis, inner ear, and most relevant to pain, the ependymal surface of the brain ventricles (Davy 2004). GeneWeaver reports an association between *Hydin* and three pain-related phenotypes: acetaminophen interaction, response to morphine, and increased inflammatory response (GS169774, GS127240, GS36729, GS35547). SPELL analysis revealed no predicted co-expressed genes for *Hydin*, and IPA returned no known functional interactions between *Hydin* and known pain genes. MouseMap predicted 2,661 genes having direct functional relationships with *Hydin* in the brain ventricular zone. VLAD analysis of the gene set revealed a high enrichment for GO terms related to reproduction, organismal development, and pattern specification—observations supported by experimental evidence for *Hydin*’s role in developmental processes (Davy 2004) and the widely acknowledged importance of motile cilia in tissue pattern specification during development (Ibanez-Tallon et al. 2003). VLAD results also revealed a highly significant enrichment for pain-related GO and MP terms, including terms related to pain threshold, behavioral pain response, thermoception, and thermal nociception. Approximately 15 % of the genes in the set had pain-related annotations, a far greater percentage than would be expected by random chance (data not shown). A significant enrichment for the pain-related GO terms “catecholamine uptake involved in synaptic transmission” (GO:0051940; *p* val = 4.93E−03) and “voltage-gated cation channel activity” (GO:0022843; *p* val = 1.53E−06) were also observed.

### Real-time qPCR analysis of *Hydin*

In order to test for differences in *Hydin* transcript levels between A/J and C57BL/6J mice, total RNA was extracted from whole brain tissue and reverse transcribed for real-time qPCR analysis of gene expression. Linear regression analysis identified strain as the only significant model term with C57BL/6J mice exhibiting significantly higher *Hydin* transcript levels compared to A/J (1.16 ± 0.056 vs. 0.99 ± 0.053, *p* = 0.0451).

The brains of C57BL/6J mice are, on average, slightly larger than those of A/J mice (Wahlsten et al. 2006), which may account for the observed differences in *Hydin* transcript levels. In the brain, *Hydin* is expressed solely in the motile cilia of the CP tissue lining the brain ventricles. CP tissue is difficult to dissect completely and exclusively. We chose to examine RNA extracted from whole brains, as we expect the amount of *Hydin* transcript from a whole brain sample to be similar to the amount observed in a perfectly dissected CP sample. While this assumption holds true for each individual sample to the best of current biological knowledge, it does not account for the fact that a mouse with a larger brain may have a greater surface area of CP tissue, and thus perhaps a greater number of motile cilia expressing *Hydin*. Therefore, we expect a lower concentration of *Hydin* in mice with larger brains. By normalizing RNA concentration to brain size, we account for the possibility that observed *Hydin* expression differences may be due to greater CP tissue volume alone. In an effort to adjust for CP tissue volume differences between samples, we normalized *Hydin* expression based on sex- and strain-specific mean brain weight (g) (Wahlsten et al. 2006; Wahlsten and Crabbe 2013). After normalizing to brain weight, we found that no model factors (sex, strain, or their interaction) were returned as significant (sex by strain interaction, *p* = 0.93; strain, *p* = 0.97; sex, *p* = 0.81).

## Discussion

We have presented here the first application of DO mice to pain genetics research. Genetic linkage mapping in DO mice produced a much more precise and efficient mapping result than conventional mapping populations. The ability to map a 3.8 Mbp QTL is a marked improvement over conventional two-strain mapping cross studies, which typically result in pain-related confidence intervals of ~30 Mbp. Precise genetic mapping combined with integrative bioinformatic techniques allowed us to identify a single candidate pain gene, *Hydin*, in a single 6 month study—an endeavor that typically requires multiple mapping and fine-mapping studies over a period of years.

The suite of bioinformatics tools described in this report outlines a systematic strategy for QTL refinement. The time- and labor-intensive nature of functional characterization studies indicates the need for computational assessment of QTL validity prior to experimental follow-up. We evaluated the genomic sufficiency of the *Tpnr6* locus by examining the SNP variants and allelic effect patterns present within the interval, allowing us to narrow *Tpnr6* to the candidate gene *Hydin*. We then assessed *Hydin*’s functional sufficiency by compiling functional annotation data summarizing *Hydin*’s current relevance to pain response. While none of the tools described here should be used exhaustively or in an exclusionary manner, all were helpful in assessing the pain-related validity of the *Tpnr6* locus.

While inbred laboratory strains exhibit sufficient genetic and phenotypic variation for behavioral QTL detection, it has been speculated that historical inbreeding processes selected for ease of handling. As previously reported by Philip et al. (2011), domestication of inbred strains, including the eight DO progenitors, likely operated on multiple loci throughout the genome. The different docility and wildness alleles present at these loci may have influenced the hot-plate endpoint behaviors measured during this study, raising the question of whether DO mice are amenable to behavioral testing. In 2011, Koide et al. (2011) reported that wild-derived inbred lines are amenable to standard behavioral testing. Since mice exhibiting atypical or “inappropriate” behaviors during behavioral testing such as repeated jumping, wall climbing, and alternate paw lifting were excluded from the present study, results from DO mice do not indicate qualitatively different behaviors in mice with wild-derived alleles at the *Tpnr6* locus, and are consistent with a study by Chesler et al. (2013) and Philip et al. (2011) that examined hot-plate response in 62 strains of mice, including the three wild-derived DO progenitors. We interpret our results to indicate that the historical inbreeding in commonly used mapping populations has inadvertently reduced the genetic and phenotypic variation available for studies of hot-plate behavior. A clear advantage of DO mice is that their heterozygous nature allows for the segregation of “lost” alleles back into the mapping population, thereby providing a broader quantitative distribution of behavior.

The suggestive QTL peak on chromosome 12 overlaps the previously identified chemical/inflammatory pain QTL *Nociq3*. *Nociq3* is 28 Mbp wide and its underlying candidate gene has not yet been elucidated. Positionally overlaying our suggestive chromosome 12 QTL onto the *Nociq3* locus provides a means for narrowing the *Nociq3* interval; the overlapping region can be taken to have the greatest probability of containing a pain-related gene, thus reducing the number of positional candidates. Overlaying our chromosome 12 locus onto *Nociq3* reduces the number of *Nociq3* candidate genes from 492 to 72 (85 %). If a mutual candidate gene was found to underlie both loci, functional characterization of the gene and its related molecular genetic pathways could shed light on nociceptive networks common to both thermal and inflammatory pain. Allelic information for the *Nociq3* locus is limited, as the study was conducted using three (C57BL/6 × MOLF/Ei) congenic strains. The genetic diversity afforded by the DO could contribute valuable allelic information to the genetic characterization of a shared candidate gene.

*Hydin* is a structural protein critical for motility in secondary cilia. Cilia are highly conserved hair-like structures generally comprised of an axoneme consisting of nine outer doublet microtubules. Motile cilia typically possess radial spokes, inner and outer dynein arms on the doublet microtubules, and a central pair of two singlet microtubules. *Hydin* is an axonemal protein, helping to position the central pair microtubules in motile cilia (Dawe et al. 2007). The synchronous beating of cilia on the ependymal surface of brain ventricles is responsible for the circulation of CSF throughout the central nervous system (CNS) (Breunig et al. 2010; Yamadori and Nara 1979). The CSF and the tissue that secrete it, the CP, have traditionally been thought to provide three primary functions within the CNS: (1) providing the brain with essential nutrients, (2) removing neuronal activity waste products from the CNS, and (3) providing mechanical support for the brain itself (Skipor and Thiery 2008). Recent evidence, however, indicates that the CP–CSF system plays a major functional role in CNS signaling. Numerous studies have demonstrated the importance of the CP–CSF system in neuroendocrine signaling within the brain (see (Skipor and Thiery 2008) for a review), suggesting that the CP–CSF system may also play a role in neuroendocrine mediated processes such as pain sensitivity and response. At the time of this writing, the CP–CSF system has only been loosely associated with pain through its known involvement in inflammatory response (Marques et al. 2009). It is known that heat sensitivity is fundamental to the experience of inflammatory pain, and recent studies have revealed genetic correlations between baseline thermal nociception and the hypersensitivity states associated with inflammatory and neuropathic pain (Mogil et al. 2005b).

The results of our plausibility analysis implicate *Hydin* in biological processes relating to pain and inflammation, suggesting the relevance of *Hydin* not only to acute thermal pain but also to inflammatory and central sensitization-mediated chronic pain as well, perhaps through involvement of the CP–CSF system. To this end, we investigated the expression levels of *Hydin* in the strains of mice reported to contribute pain-sensitive (C57BL/6J) and pain-resistant (A/J) alleles at the *Tpnr6* locus using real-time quantitative PCR. After normalizing to brain weight, no *Hydin* expression differences were observed between the two strains. The murine *Hydin* gene contains 1100 SNPs: 33 coding-synonymous, 8 coding-nonsynonymous, 1059 intronic (dbSNP Build 128, http://www.ncbi.nlm.nih.gov/projects/SNP/; NCBI Build 37). Only seven *Hydin* SNPs differ between A/J and C57BL/6J, all of which are located in intronic sequence (rs32704439, rs13479984, rs33400842, rs33170521, rs32685085, rs33110413, rs32952051). There are three SNPs located in *Hydin* regulatory regions: two proximal upstream SNPs located in the predicted gene *Gm17466* (rs50054724 and rs49903084) and one downstream SNP located 1,111 bp downstream of *Hydin* (rs31253280), but there are no upstream regulatory polymorphisms between A/J and C57BL/6J. The Wellcome Trust Sanger Institute (REL-1211; NCBI build 37) reports four CAST/EiJ-exclusive SNPs present in *Hydin* splice regions (rs214233349, rs234089050, rs253098678, no rsID), suggesting a *Hydin* functional difference private to CAST/EiJ. These observations suggest that *Hydin* may influence pain sensitivity through mechanisms other than functional polymorphisms regulating gene expression, such as differences in mRNA splicing.

The precise mechanism(s) by which *Hydin* influences hot-plate sensitivity are not known. It is most likely that CSF circulation of endogenous peptides plays a role, thereby establishing pain as a ciliopathic condition. However, indirect mechanisms are also possible; for example, *Hydin* may influence hot-plate behavior through biological mechanisms related to stress, locomotion, or other behavioral phenotypes. Future studies to perform deeper functional characterization of the role of *Hydin* and the particular SNPs discussed in this report will be needed to confirm *Hydin*’s functional role in conditioning pain sensitivity. Through the use of QTL mapping in the Diversity Outcross population we were able to rapidly identify multiple genetic variants that may modify the activity of *Hydin* and its role in pain sensitivity in an intact mammalian system.
